# Patterns of oral anticoagulant use and outcomes in Asian patients with atrial fibrillation: a post-hoc analysis from the GLORIA-AF Registry

**DOI:** 10.1016/j.eclinm.2023.102039

**Published:** 2023-08-25

**Authors:** Giulio Francesco Romiti, Bernadette Corica, Marco Proietti, Davide Antonio Mei, Juliane Frydenlund, Arnaud Bisson, Giuseppe Boriani, Brian Olshansky, Yi-Hsin Chan, Menno V. Huisman, Tze-Fan Chao, Gregory Y.H. Lip, GLORIA-AF Investigators, Dzifa Wosornu Abban, Nasser Abdul, Atilio Marcelo Abud, Fran Adams, Srinivas Addala, Pedro Adragão, Walter Ageno, Rajesh Aggarwal, Sergio Agosti, Piergiuseppe Agostoni, Francisco Aguilar, Julio Aguilar Linares, Luis Aguinaga, Jameel Ahmed, Allessandro Aiello, Paul Ainsworth, Jorge Roberto Aiub, Raed Al-Dallow, Lisa Alderson, Jorge Antonio Aldrete Velasco, Dimitrios Alexopoulos, Fernando Alfonso Manterola, Pareed Aliyar, David Alonso, Fernando Augusto Alves da Costa, José Amado, Walid Amara, Mathieu Amelot, Nima Amjadi, Fabrizio Ammirati, Marianna Andrade, Nabil Andrawis, Giorgio Annoni, Gerardo Ansalone, M.Kevin Ariani, Juan Carlos Arias, Sébastien Armero, Chander Arora, Muhammad Shakil Aslam, M. Asselman, Philippe Audouin, Charles Augenbraun, S. Aydin, Ivaneta Ayryanova, Emad Aziz, Luciano Marcelo Backes, E. Badings, Ermentina Bagni, Seth H. Baker, Richard Bala, Antonio Baldi, Shigenobu Bando, Subhash Banerjee, Alan Bank, Gonzalo Barón Esquivias, Craig Barr, Maria Bartlett, Vanja Basic Kes, Giovanni Baula, Steffen Behrens, Alan Bell, Raffaella Benedetti, Juan Benezet Mazuecos, Bouziane Benhalima, Jutta Bergler-Klein, Jean-Baptiste Berneau, Richard A. Bernstein, Percy Berrospi, Sergio Berti, Andrea Berz, Elizabeth Best, Paulo Bettencourt, Robert Betzu, Ravi Bhagwat, Luna Bhatta, Francesco Biscione, Giovanni Bisignani, Toby Black, Michael J. Bloch, Stephen Bloom, Edwin Blumberg, Mario Bo, Ellen Bøhmer, Andreas Bollmann, Maria Grazia Bongiorni, Giuseppe Boriani, D.J. Boswijk, Jochen Bott, Edo Bottacchi, Marica Bracic Kalan, Drew Bradman, Donald Brautigam, Nicolas Breton, P.J.A.M. Brouwers, Kevin Browne, Jordi Bruguera Cortada, A. Bruni, Claude Brunschwig, Hervé Buathier, Aurélie Buhl, John Bullinga, Jose Walter Cabrera, Alberto Caccavo, Shanglang Cai, Sarah Caine, Leonardo Calò, Valeria Calvi, Mauricio Camarillo Sánchez, Rui Candeias, Vincenzo Capuano, Alessandro Capucci, Ronald Caputo, Tatiana Cárdenas Rizo, Francisco Cardona, Francisco Carlos da Costa Darrieux, Yan Carlos Duarte Vera, Antonio Carolei, Susana Carreño, Paula Carvalho, Susanna Cary, Gavino Casu, Claudio Cavallini, Guillaume Cayla, Aldo Celentano, Tae-Joon Cha, Kwang Soo Cha, Jei Keon Chae, Kathrine Chalamidas, Krishnan Challappa, Sunil Prakash Chand, Harinath Chandrashekar, Ludovic Chartier, Kausik Chatterjee, Carlos Antero Chavez Ayala, Aamir Cheema, Amjad Cheema, Lin Chen, Shih-Ann Chen, Jyh Hong Chen, Fu-Tien Chiang, Francesco Chiarella, Lin Chih-Chan, Yong Keun Cho, Jong-Il Choi, Dong Ju Choi, Guy Chouinard, Danny Hoi-Fan Chow, Dimitrios Chrysos, Galina Chumakova, Eduardo Julián José Roberto, Chuquiure Valenzuela, Nicoleta Cindea Nica, David J. Cislowski, Anthony Clay, Piers Clifford, Andrew Cohen, Michael Cohen, Serge Cohen, Furio Colivicchi, Ronan Collins, Paolo Colonna, Steve Compton, Derek Connolly, Alberto Conti, Gabriel Contreras Buenostro, Gregg Coodley, Martin Cooper, Julian Coronel, Giovanni Corso, Juan Cosín Sales, Yves Cottin, John Covalesky, Aurel Cracan, Filippo Crea, Peter Crean, James Crenshaw, Tina Cullen, Harald Darius, Patrick Dary, Olivier Dascotte, Ira Dauber, Vicente Davalos, Ruth Davies, Gershan Davis, Jean-Marc Davy, Mark Dayer, Marzia De Biasio, Silvana De Bonis, Raffaele De Caterina, Teresiano De Franceschi, J.R. de Groot, José De Horta, Axel De La Briolle, Gilberto de la Pena Topete, Angelo Amato Vicenzo de Paola, Weimar de Souza, A. de Veer, Luc De Wolf, Eric Decoulx, Sasalu Deepak, Pascal Defaye, Freddy Del-Carpio Munoz, Diana Delic Brkljacic, N. Joseph Deumite, Silvia Di Legge, Igor Diemberger, Denise Dietz, Pedro Dionísio, Qiang Dong, Fabio Rossi dos Santos, Elena Dotcheva, Rami Doukky, Anthony D'Souza, Simon Dubrey, Xavier Ducrocq, Dmitry Dupljakov, Mauricio Duque, Dipankar Dutta, Nathalie Duvilla, A. Duygun, Rainer Dziewas, Charles B. Eaton, William Eaves, L.A. Ebels-Tuinbeek; Clifford Ehrlich, Sabine Eichinger-Hasenauer, Steven J. Eisenberg, Adnan El Jabali, Mahfouz El Shahawy, Mauro Esteves Hernandes, Ana Etxeberria Izal, Rudolph Evonich, Oksana Evseeva, Andrey Ezhov, Raed Fahmy, Quan Fang, Ramin Farsad, Laurent Fauchier, Stefano Favale, Maxime Fayard, Jose Luis Fedele, Francesco Fedele, Olga Fedorishina, Steven R. Fera, Luis Gustavo Gomes Ferreira, Jorge Ferreira, Claudio Ferri, Anna Ferrier, Hugo Ferro, Alexandra Finsen, Brian First, Stuart Fischer, Catarina Fonseca, Luísa Fonseca Almeida, Steven Forman, Brad Frandsen, William French, Keith Friedman, Athena Friese, Ana Gabriela Fruntelata, Shigeru Fujii, Stefano Fumagalli, Marta Fundamenski, Yutaka Furukawa, Matthias Gabelmann, Nashwa Gabra, Niels Gadsbøll, Michel Galinier, Anders Gammelgaard, Priya Ganeshkumar, Christopher Gans, Antonio Garcia Quintana, Olivier Gartenlaub, Achille Gaspardone, Conrad Genz, Frédéric Georger, Jean-Louis Georges, Steven Georgeson, Evaldas Giedrimas, Mariusz Gierba, Ignacio Gil Ortega, Eve Gillespie, Alberto Giniger, Michael C. Giudici, Alexandros Gkotsis, Taya V. Glotzer, Joachim Gmehling, Jacek Gniot, Peter Goethals, Seth Goldbarg, Ronald Goldberg, Britta Goldmann, Sergey Golitsyn, Silvia Gómez, Juan Gomez Mesa, Vicente Bertomeu Gonzalez, Jesus Antonio Gonzalez Hermosillo, Víctor Manuel González López, Hervé Gorka, Charles Gornick, Diana Gorog, Venkat Gottipaty, Pascal Goube, Ioannis Goudevenos, Brett Graham, G. Stephen Greer, Uwe Gremmler, Paul G. Grena, Martin Grond, Edoardo Gronda, Gerian Grönefeld, Xiang Gu, Ivett Guadalupe Torres Torres, Gabriele Guardigli, Carolina Guevara, Alexandre Guignier, Michele Gulizia, Michael Gumbley, Albrecht Günther, Andrew Ha, Georgios Hahalis, Joseph Hakas, Christian Hall, Bing Han, Seongwook Han, Joe Hargrove, David Hargroves, Kenneth B. Harris, Tetsuya Haruna, Emil Hayek, Jeff Healey, Steven Hearne, Michael Heffernan, Geir Heggelund, J.A. Heijmeriks, Maarten Hemels, I. Hendriks, Sam Henein, Sung-Ho Her, Paul Hermany, Jorge Eduardo Hernández Del Río, Yorihiko Higashino, Michael Hill, Tetsuo Hisadome, Eiji Hishida, Etienne Hoffer, Matthew Hoghton, Kui Hong, Suk keun Hong, Stevie Horbach, Masataka Horiuchi, Yinglong Hou, Jeff Hsing, Chi-Hung Huang, David Huckins, kathy Hughes, A. Huizinga, E.L. Hulsman, Kuo-Chun Hung, Gyo-Seung Hwang, Margaret Ikpoh, Davide Imberti, Hüseyin Ince, Ciro Indolfi, Shujiro Inoue, Didier Irles, Harukazu Iseki, C. Noah Israel, Bruce Iteld, Venkat Iyer, Ewart Jackson-Voyzey, Naseem Jaffrani, Frank Jäger, Martin James, Sung-Won Jang, Nicolas Jaramillo, Nabil Jarmukli, Robert J. Jeanfreau, Ronald D. Jenkins, Carlos Jerjes Sánchez, Javier Jimenez, Robert Jobe, Tomas Joen-Jakobsen, Nicholas Jones, Jose Carlos Moura Jorge, Bernard Jouve, Byung Chun Jung, Kyung Tae Jung, Werner Jung, Mikhail Kachkovskiy, Krystallenia Kafkala, Larisa Kalinina, Bernd Kallmünzer, Farzan Kamali, Takehiro Kamo, Priit Kampus, Hisham Kashou, Andreas Kastrup, Apostolos Katsivas, Elizabeth Kaufman, Kazuya Kawai, Kenji Kawajiri, John F. Kazmierski, P. Keeling, José Francisco Kerr Saraiva, Galina Ketova, Ajit Singh Khaira, Aleksey Khripun, Doo-Il Kim, Young Hoon Kim, Nam Ho Kim, Dae Kyeong Kim, Jeong Su Kim, June Soo Kim, Ki Seok Kim, Jin bae Kim, Elena Kinova, Alexander Klein, James J. Kmetzo, G. Larsen Kneller, Aleksandar Knezevic, Su Mei Angela Koh, Shunichi Koide, Anastasios Kollias, J.A. Kooistra, Jay Koons, Martin Koschutnik, William J. Kostis, Dragan Kovacic, Jacek Kowalczyk, Natalya Koziolova, Peter Kraft, Johannes A. Kragten, Mori Krantz, Lars Krause, B.J. Krenning, F. Krikke, Z. Kromhout, Waldemar Krysiak, Priya Kumar, Thomas Kümler, Malte Kuniss, Jen-Yuan Kuo, Achim Küppers, Karla Kurrelmeyer, Choong Hwan Kwak, Bénédicte Laboulle, Arthur Labovitz, Wen Ter Lai, Andy Lam, Yat Yin Lam, Fernando Lanas Zanetti, Charles Landau, Giancarlo Landini, Estêvão Lanna Figueiredo, Torben Larsen, Karine Lavandier, Jessica LeBlanc, Moon Hyoung Lee, Chang-Hoon Lee, John Lehman, Ana Leitão, Nicolas Lellouche, Malgorzata Lelonek, Radoslaw Lenarczyk, T. Lenderink, Salvador León González, Peter Leong-Sit, Matthias Leschke, Nicolas Ley, Zhanquan Li, Xiaodong Li, Weihua Li, Xiaoming Li, Christhoh Lichy, Ira Lieber, Ramon Horacio Limon Rodriguez, Hailong Lin, Gregory Y.H. Lip, Feng Liu, Hengliang Liu, Guillermo Llamas Esperon, Nassip Llerena Navarro, Eric Lo, Sergiy Lokshyn, Amador López, José Luís López-Sendón, Adalberto Menezes Lorga Filho, Richard S. Lorraine, Carlos Alberto Luengas, Robert Luke, Ming Luo, Steven Lupovitch, Philippe Lyrer, Changsheng Ma, Genshan Ma, Irene Madariaga, Koji Maeno, Dominique Magnin, Gustavo Maid, Sumeet K. Mainigi, Konstantinos Makaritsis, Rohit Malhotra, Rickey Manning, Athanasios Manolis, Helard Andres Manrique Hurtado, Ioannis Mantas, Fernando Manzur Jattin, Vicky Maqueda, Niccolo Marchionni, Francisco Marin Ortuno, Antonio Martín Santana, Jorge Martinez, Petra Maskova, Norberto Matadamas Hernandez, Katsuhiro Matsuda, Tillmann Maurer, Ciro Mauro, Erik May, Nolan Mayer, John McClure, Terry McCormack, William McGarity, Hugh McIntyre, Brent McLaurin, Feliz Alvaro Medina Palomino, Francesco Melandri, Hiroshi Meno, Dhananjai Menzies, Marco Mercader, Christian Meyer, Beat J. Meyer, Jacek Miarka, Frank Mibach, Dominik Michalski, Patrik Michel, Rami Mihail Chreih, Ghiath Mikdadi, Milan Mikus, Davor Milicic, Constantin Militaru, Sedi Minaie, Bogdan Minescu, Iveta Mintale, Tristan Mirault, Michael J. Mirro, Dinesh Mistry, Nicoleta Violeta Miu, Naomasa Miyamoto, Tiziano Moccetti, Akber Mohammed, Azlisham Mohd Nor, Michael Mollerus, Giulio Molon, Sergio Mondillo, Patrícia Moniz, Lluis Mont, Vicente Montagud, Oscar Montaña, Cristina Monti, Luciano Moretti, Kiyoo Mori, Andrew Moriarty, Jacek Morka, Luigi Moschini, Nikitas Moschos, Andreas Mügge, Thomas J. Mulhearn, Carmen Muresan, Michela Muriago, Wlodzimierz Musial, Carl W. Musser, Francesco Musumeci, Thuraia Nageh, Hidemitsu Nakagawa, Yuichiro Nakamura, Toru Nakayama, Gi-Byoung Nam, Michele Nanna, Indira Natarajan, Hemal M. Nayak, Stefan Naydenov, Jurica Nazlić, Alexandru Cristian Nechita, Libor Nechvatal, Sandra Adela Negron, James Neiman, Fernando Carvalho Neuenschwander, David Neves, Anna Neykova, Ricardo Nicolás Miguel, George Nijmeh, Alexey Nizov, Rodrigo Noronha Campos, Janko Nossan, Tatiana Novikova, Ewa Nowalany-Kozielska, Emmanuel Nsah, Juan Carlos Nunez Fragoso, Svetlana Nurgalieva, Dieter Nuyens, Ole Nyvad, Manuel Odin de Los Rios Ibarra, Philip O'Donnell, Martin O'Donnell, Seil Oh, Yong Seog Oh, Dongjin Oh, Gilles O'Hara, Kostas Oikonomou, Claudia Olivares, Richard Oliver, Rafael Olvera Ruiz, Christoforos Olympios, Anna omaszuk-Kazberuk, Joaquín Osca Asensi, eena Padayattil Jose, Francisco Gerardo Padilla Padilla, Victoria Padilla Rios, Giuseppe Pajes, Shekhar Pandey, Gaetano Paparella, F. Paris, Hyung Wook Park, Jong Sung Park, Fragkiskos Parthenakis, Enrico Passamonti, Rajesh J. Patel, Jaydutt Patel, Mehool Patel, Janice Patrick, Ricardo Pavón Jimenez, Analía Paz, Vittorio Pengo, William Pentz, Beatriz Pérez, Alma Minerva Pérez Ríos, Alejandro Pérez-Cabezas, Richard Perlman, Viktor Persic, Francesco Perticone, Terri K. Peters, Sanjiv Petkar, Luis Felipe Pezo, Christian Pflücke, David N. Pham, Roland T. Phillips, Stephen Phlaum, Denis Pieters, Julien Pineau, Arnold Pinter, Fausto Pinto, R. Pisters, Nediljko Pivac, Darko Pocanic, Cristian Podoleanu, Alessandro Politano, Zdravka Poljakovic, Stewart Pollock, Jose Polo Garcéa, Holger Poppert, Maurizio Porcu, Antonio Pose Reino, Neeraj Prasad, Dalton Bertolim Précoma, Alessandro Prelle, John Prodafikas, Konstantin Protasov, Maurice Pye, Zhaohui Qiu, Jean-Michel Quedillac, Dimitar Raev, Carlos Antonio Raffo Grado, Sidiqullah Rahimi, Arturo Raisaro, Bhola Rama, Ricardo Ramos, Maria Ranieri, Nuno Raposo, Eric Rashba, Ursula Rauch-Kroehnert, Ramakota Reddy, Giulia Renda, Shabbir Reza, Luigi Ria, Dimitrios Richter, Hans Rickli, Werner Rieker, Tomas Ripolil Vera, Luiz Eduardo Ritt, Douglas Roberts, Ignacio Rodriguez Briones, Aldo Edwin Rodriguez Escudero, Carlos Rodríguez Pascual, Mark Roman, Francesco Romeo, E. Ronner, Jean-Francois Roux, Nadezda Rozkova, Miroslav Rubacek, Frank Rubalcava, Andrea M. Russo, Matthieu Pierre Rutgers, Karin Rybak, Samir Said, Tamotsu Sakamoto, Abraham Salacata, Adrien Salem, Rafael Salguero Bodes, Marco A. Saltzman, Alessandro Salvioni, Gregorio Sanchez Vallejo, Marcelo Sanmartín Fernández, Wladmir Faustino Saporito, Kesari Sarikonda, Taishi Sasaoka, Hamdi Sati, Irina Savelieva, Pierre-Jean Scala, Peter Schellinger, Carlos Scherr, Lisa Schmitz, Karl-Heinz Schmitz, Bettina Schmitz, Teresa Schnabel, Steffen Schnupp, Peter Schoeniger, Norbert Schön, Peter Schwimmbeck, Clare Seamark, Greg Searles, Karl-Heinz Seidl, Barry Seidman, Jaroslaw Sek, Lakshmanan Sekaran, Carlo Serrati, Neerav Shah, Vinay Shah, Anil Shah, Shujahat Shah, Vijay Kumar Sharma, Louise Shaw, Khalid H. Sheikh, Naruhito Shimizu, Hideki Shimomura, Dong-Gu Shin, Eun-Seok Shin, Junya Shite, Gerolamo Sibilio, Frank Silver, Iveta Sime, Tim A. Simmers, Narendra Singh, Peter Siostrzonek, Didier Smadja, David W. Smith, Marcelo Snitman, Dario Sobral Filho, Hassan Soda, Carl Sofley, Adam Sokal, Yannie Soo Oi Yan, Rodolfo Sotolongo, Olga Ferreira de Souza, Jon Arne Sparby, Jindrich Spinar, David Sprigings, Alex C. Spyropoulos, Dimitrios Stakos, Clemens Steinwender, Georgios Stergiou, Ian Stiell, Marcus Stoddard, Anastas Stoikov, Witold Streb, Ioannis Styliadis, Guohai Su, Xi Su, Wanda Sudnik, Kai Sukles, Xiaofei Sun, H. Swart, Janko Szavits-Nossan, Jens Taggeselle, Yuichiro Takagi, Amrit Pal Singh Takhar, Angelika Tamm, Katsumi Tanaka, Tanyanan Tanawuttiwat, Sherman Tang, Aylmer Tang, Giovanni Tarsi, Tiziana Tassinari, Ashis Tayal, Muzahir Tayebjee, J.M. ten Berg, Dan Tesloianu, Salem H.K. The, Dierk Thomas, Serge Timsit, Tetsuya Tobaru, Andrzej R. Tomasik, Mikhail Torosoff, Emmanuel Touze, Elina Trendafilova, W. Kevin Tsai, Hung Fat Tse, Hiroshi Tsutsui, Tian Ming Tu, Ype Tuininga, Minang Turakhia, Samir Turk, Wayne Turner, Arnljot Tveit, Richard Tytus, C. Valadão, P.F.M.M. van Bergen, Philippe van de Borne, B.J. van den Berg, C. van der Zwaan, M. Van Eck, Peter Vanacker, Dimo Vasilev, Vasileios Vasilikos, Maxim Vasilyev, Srikar Veerareddy, Mario Vega Miño; Asok Venkataraman, Paolo Verdecchia, Francesco Versaci, Ernst Günter Vester, Hubert Vial, Jason Victory, Alejandro Villamil, Marc Vincent, Anthony Vlastaris, Jürgen vom Dahl, Kishor Vora, Robert B. Vranian, Paul Wakefield, Ningfu Wang, Mingsheng Wang, Xinhua Wang, Feng Wang, Tian Wang, Alberta L. Warner, Kouki Watanabe, Jeanne Wei, Christian Weimar, Stanislav Weiner, Renate Weinrich, Ming-Shien Wen, Marcus Wiemer, Preben Wiggers, Andreas Wilke, David Williams, Marcus L. Williams, Bernhard Witzenbichler, Brian Wong, Ka Sing Lawrence Wong, Beata Wozakowska-Kaplon, Shulin Wu, Richard C. Wu, Silke Wunderlich, Nell Wyatt, John (Jack) Wylie, Yong Xu, Xiangdong Xu, Hiroki Yamanoue, Takeshi Yamashita, Ping Yen Bryan Yan, Tianlun Yang, Jing Yao, Kuo-Ho Yeh, Wei Hsian Yin, Yoto Yotov, Ralf Zahn, Stuart Zarich, Sergei Zenin, Elisabeth Louise Zeuthen, Huanyi Zhang, Donghui Zhang, Xingwei Zhang, Ping Zhang, Jun Zhang, Shui Ping Zhao, Yujie Zhao, Zhichen Zhao, Yang Zheng, Jing Zhou, Sergio Zimmermann, Andrea Zini, Steven Zizzo, Wenxia Zong, L. Steven Zukerman

**Affiliations:** a Liverpool Centre for Cardiovascular Sciences at University of Liverpool, Liverpool John Moores University and Liverpool Heart & Chest Hospital, Liverpool, United Kingdom; b Department of Translational and Precision Medicine, Sapienza – University of Rome, Rome, Italy; c Department of Clinical Sciences and Community Health, University of Milan, Milan, Italy; d Geriatric Unit, IRCCS Istituti Clinici Scientifici Maugeri, Milan, Italy; e Cardiology Division, Department of Biomedical, Metabolic and Neural Sciences, University of Modena and Reggio Emilia, Policlinico di Modena, Modena, Italy; f Danish Center for Clinical Health Services Research, Aalborg University, Aalborg, Denmark; g Service de Cardiologie, CHU Trousseau et Université François Rabelais, Tours, France; h Division of Cardiology, Department of Medicine, University of Iowa, Iowa City, USA; i Cardiovascular Department, Chang Gung Memorial Hospital, Linkou, Taoyuan City, Taiwan; j College of Medicine, Chang Gung University, Taoyuan City, Taiwan; k Microscopy Core Laboratory, Chang Gung Memorial Hospital, Linkou, Taoyuan City Taiwan; l Department of Thrombosis and Hemostasis, Leiden University Medical Center, Leiden, the Netherlands; m Division of Cardiology, Department of Medicine, Taipei Veterans General Hospital, Taipei, Taiwan; n Institute of Clinical Medicine, and Cardiovascular Research Center, National Yang Ming Chiao Tung University, Taipei, Taiwan

**Keywords:** Atrial fibrillation, Ethnic differences, Asia, Oral anticoagulant, Outcomes

## Abstract

**Background:**

Previous studies suggested potential ethnic differences in the management and outcomes of atrial fibrillation (AF). We aim to analyse oral anticoagulant (OAC) prescription, discontinuation, and risk of adverse outcomes in Asian patients with AF, using data from a global prospective cohort study.

**Methods:**

From the GLORIA-AF Registry Phase II–III (November 2011–December 2014 for Phase II, and January 2014–December 2016 for Phase III), we analysed patients according to their self-reported ethnicity (Asian vs. non-Asian), as well as according to Asian subgroups (Chinese, Japanese, Korean and other Asian). Logistic regression was used to analyse OAC prescription, while the risk of OAC discontinuation and adverse outcomes were analysed through Cox-regression model. Our primary outcome was the composite of all-cause death and major adverse cardiovascular events (MACE). The original studies were registered with ClinicalTrials.gov, NCT01468701, NCT01671007, and NCT01937377.

**Findings:**

34,421 patients were included (70.0 ± 10.5 years, 45.1% females, 6900 (20.0%) Asian: 3829 (55.5%) Chinese, 814 (11.8%) Japanese, 1964 (28.5%) Korean and 293 (4.2%) other Asian). Most of the Asian patients were recruited in Asia (n = 6701, 97.1%), while non-Asian patients were mainly recruited in Europe (n = 15,449, 56.1%) and North America (n = 8378, 30.4%). Compared to non-Asian individuals, prescription of OAC and non-vitamin K antagonist oral anticoagulant (NOAC) was lower in Asian patients (Odds Ratio [OR] and 95% Confidence Intervals (CI): 0.23 [0.22–0.25] and 0.66 [0.61–0.71], respectively), but higher in the Japanese subgroup. Asian ethnicity was also associated with higher risk of OAC discontinuation (Hazard Ratio [HR] and [95% CI]: 1.79 [1.67–1.92]), and lower risk of the primary composite outcome (HR [95% CI]: 0.86 [0.76–0.96]). Among the exploratory secondary outcomes, Asian ethnicity was associated with higher risks of thromboembolism and intracranial haemorrhage, and lower risk of major bleeding.

**Interpretation:**

Our results showed that Asian patients with AF showed suboptimal thromboembolic risk management and a specific risk profile of adverse outcomes; these differences may also reflect differences in country-specific factors. Ensuring integrated and appropriate treatment of these patients is crucial to improve their prognosis.

**Funding:**

The GLORIA-AF Registry was funded by 10.13039/100001003Boehringer Ingelheim GmbH.

**Research in context**

**Evidence before this study**

We searched PubMed from inception to April 30, 2023, without language restrictions, for randomised trials, systematic reviews, meta-analyses, and observational studies, using the terms “Atrial Fibrillation” and “Asian”. Ethnic differences have been described in patients with Atrial Fibrillation, with Asian individuals found more frequently undertreated with oral anticoagulant, and with different risk of long-term outcomes. Epidemiological data, and our understanding of the differences between Asian and non-Asian patients with AF, are still limited.

**Added value of this study**

In this large analysis including more than 34,000 patients with AF, we found that Asian ethnicity was associated with lower odds of oral anticoagulant prescription, higher risk of treatment discontinuation, and an overall different prognosis (with lower risk of the primary composite outcome of all-cause death and major adverse cardiovascular events, but higher risk of thromboembolism and intracerebral haemorrhage).

**Implications of all the available evidence**

Asian patients with AF are often suboptimally managed for thromboembolic risk prevention, and show a specific prognostic risk profile. Acknowledging ethnic differences in the natural history of AF is pivotal to ensure equal and optimal care and to improve outcomes of AF patients.

## Introduction

With progressive ageing of the population, the incidence of AF is increasing, with prevalence projected to reach over 17 million individuals in Europe and 72 million in Asia by 2050.^1^^,^^2^ Despite improvements in the treatment of AF, the risks of adverse outcomes and healthcare costs are still high.^3^, ^4^, ^5^

Importantly, regional differences in the epidemiology and natural history of AF are increasingly reported, including different age at onset, and predisposition to AF-related adverse events.^6^ Asian countries report a lower prevalence of AF compared to Western ones, although the risk of thromboembolic events and intracranial bleeding seems higher in Asian patients.^6^, ^7^, ^8^, ^9^

The large-scale uptake of non-vitamin K antagonist oral anticoagulants (NOACs) in clinical practice have progressively led to a paradigm shift for stroke prevention of patients with AF, and data from post-hoc analysis of randomised controlled trials have shown that NOACs may provide significant benefit (both in terms of efficacy and safety) in Asian patients, compared to vitamin K antagonist (VKA).^7^^,^^10^, ^11^, ^12^

However, there are still relatively limited epidemiological data on Asian patients,^8^ and previous reports have identified potential ethnic differences in the uptake of oral anticoagulant (OAC), with potential undertreatment of Asian patients.^13^ Indeed, one previous analysis from the Phase III of the prospective Global Registry on Long-Term Oral Anti-thrombotic Treatment in Patients with Atrial Fibrillation (GLORIA-AF) showed regional-based differences in OAC use among patients with AF.^14^

In this analysis, using data from both Phase II and Phase III of the GLORIA-AF Registry, we compared Asian vs. non-Asian patients with AF, in terms of OAC prescription, risk of OAC discontinuation, and incidence of major adverse events.

## Methods

### Study design

The design of GLORIA-AF Phase II and III registry have been previously reported.^15^ In short, GLORIA-AF is an international, multicentre prospective registry program structured in 3 phases, aiming at assessing the long-term effectiveness and safety of dabigatran etexilate in real-world patients with AF. During the study period (November 2011–December 2014 for Phase II, and January 2014–December 2016 for Phase III), patients with newly-diagnosed non-valvular AF and CHA_2_DS_2_-VASc score ≥1 were consecutively enrolled in the study. The primary results of the GLORIA-AF Phase III registry have already been published.^16^^,^^17^ The original studies were registered with ClinicalTrials.gov, NCT01468701, NCT01671007, and NCT01937377.

### Inclusion/exclusion criteria and procedures

Full details on inclusion and exclusion criteria are reported elsewhere.^17^ Briefly, eligible patients for the inclusion in both Phase II and Phase III of the GLORIA-AF registry were adult patients (≥18 years), with a recent diagnosis of AF (<3 months, or <4.5 months only in Latin America), and a CHA_2_DS_2_-VASc score ≥1, who provided written informed consent. Main exclusion criteria were: mechanical heart valve (or expected to undergo valve replacement); having received VKA for >60 days in the lifetime; having other clinical indication for OAC; short life expectancy (<1 year); or AF due to a reversible cause.

The study was conducted in accordance with the principles of Good Clinical Practice and the Declaration of Helsinki, and approved by local institutional review board at participating sites. At baseline, data regarding demographics (including self-reported ethnicity), comorbidities, and treatment prescribed were recorded by investigators, for each patient, in standardised electronic case report forms.

### Definition of Asian ethnicity

For this analysis, we included only those patients with available data regarding ethnicity (i.e., being Asian or not). Ethnicity was self-reported by patients and collected in the electronic case report form. Specifically, patients were able to select “Asian” among the ethnicities proposed (which included also American Indian/American Native/Alaska Native, Black/African American, White, Native Hawaiian/Other Pacific Islander, Arab/Middle East, African and Other), and if so, they were also able to sub-specify whether they were Chinese, Japanese, Korean, other Asian or South Asian. As these two last categories accounted for <1% of total patients, for purposes of this analysis, we grouped them together among “other Asian”.

For all the comparisons, the reference group was the one composed of non-Asians patients, which encompassed all other ethnicities (i.e., White, Black/African American, and others).

### Treatment, follow-up and major adverse outcomes

At the baseline, data on antithrombotic prescription was collected by investigators. Details on follow-up and outcomes for GLORIA-AF Phase II and Phase III were reported elsewhere.^17^^,^^18^ Briefly, patients who received dabigatran during Phase II were followed-up for 2 years, while all patients enrolled during the Phase III of the program were followed-up, irrespective of the antithrombotic treatment received at baseline, for 3 years. During follow-up, data regarding OAC discontinuation and major outcomes were recorded, until study withdrawal, death or end of the study. In this analysis, we evaluated non-persistence and discontinuation only for patients who received OAC at baseline. We defined OAC discontinuation as either switching to another antithrombotic regimen (including switching to a different OAC), or interruption ≥30-days of the treatment received at baseline. Dose changes were not considered discontinuation. We defined non-persistence as either discontinuation or study termination.

We also evaluated the associations between ethnicity and the risk of major outcomes occurred during follow-up. For this analysis, we defined our primary outcome as the composite of all-cause death and MACEs (defined as the occurrence of cardiovascular death, stroke, and myocardial infarction). We also investigated the following exploratory secondary outcomes:

- All-cause death;
- Cardiovascular death;
- Stroke (haemorrhagic, ischemic, and uncertain classification strokes);
- Thromboembolism (composite of stroke, transient ischemic attack (TIA), and other non-central nervous system thromboembolism),
- Major Adverse Cardiovascular Events (MACEs)
- Major bleeding (defined according to the International Society of Thrombosis and Haemostasis classification, i.e., overt bleeding associated with a haemoglobin reduction of ≥20 g/L or leading to ≥2-unit of blood transfusion, symptomatic bleeding in a critical organ, life-threatening or fatal bleeding).
- Among major bleeding events, we evaluated the subgroup of intracranial haemorrhages (ICH), defined as intracranial subdural, epidural, subarachnoid, intraventricular, intracerebral and non-specified bleeding), and non-ICH major bleedings.

### Statistical analysis

Continuous variables were reported as mean and standard deviation (SD) or median and interquartile range [IQR] and compared with appropriate parametric and non-parametric tests according to whether they were normally distributed or not, respectively. Categorical variables, reported as frequencies and percentages, were compared using chi-square test.

The associations between Asian ethnicity and prescription of OAC at baseline were analysed through multivariable logistic regression; results were reported as Odds Ratio (OR) and 95% Confidence Intervals (CI). Cox-regression models were used to evaluate the association between Asian ethnicity and both OAC discontinuation and risk of outcomes, and results were presented as Hazard Ratio (HR) and 95% CI. For both logistic and Cox regression models, we analysed two set of multivariable models: in the first (main model) we included the following covariates: age, sex, type of AF (paroxysmal, persistent or permanent), history of previous bleeding, body mass index (BMI) and CHA_2_DS_2_-VASc score; in the second (model 2), we included the same covariates of model 1, replacing CHA_2_DS_2_-VASc with the individual comorbidities which compose the score (history of stroke/TIA, heart failure, arterial hypertension, diabetes, peripheral vascular disease, and coronary artery disease). Additionally, for the analysis on major outcomes, we also adjusted models for the use of OAC. Finally, we calculated incidence rates (IR) and 95% CI according to the number of events and person-years.^19^ For the primary outcome, we reported Kaplan–Meier curves. Survival distributions were compared using Log-Rank test.

We performed additional analyses. For all outcomes investigated (OAC prescription and discontinuation, risk of major outcomes), we analysed the Asian ethnicity subgroups, as previously defined, and we also evaluated the interaction between Asian ethnicity and the phase of enrolment (Phase II vs. Phase III).

A two-sided p < 0.05 was considered statistically significant. All the analyses were performed using R 4.0.3 (R Core Team 2020, Vienna, Austria).

### Role of the funding source

The GLORIA-AF Registry was funded by Boehringer Ingelheim. The authors are solely responsible for the design and conduct of this study, all study analyses, drafting and editing of the manuscript, and its final content. GFR, BC and GYHL had access to dataset and decided to submit the manuscript. All authors supported the decision to submit the manuscript for publication.

## Results

From the 36,617 patients originally enrolled in the GLORIA-AF Phase II and III Registry, 34,421 (94.0%; mean age 70.0 (10.5) years, 15,518 (45.1%) females) with available data on self-reported ethnicity were included in this analysis. Of these, 6900 (20%) were Asian: 3829 were Chinese (55.5% of the total Asian group), 1964 Korean (28.5%), 814 Japanese (11.8%) and 293 other Asians (4.2%). Among non-Asian patients, the vast majority (91.3%) were White.

Baseline characteristics according to ethnicity (Asian vs. non-Asian) and subgroups are reported in Supplementary Table S1 and S2 in Supplementary Materials. Asian patients were younger, less likely female, showed lower BMI values, and had an overall lower burden of most comorbidities. Accordingly, baseline CHA_2_DS_2_-VASc and HAS-BLED scores were significantly lower among Asian patients.

Several differences were also observed among subgroups of Asian patients, with Japanese patients being older, and Korean patients having a lower prevalence of most comorbidities. Overall, most Asian patients were recruited in Asia (97.1%). Among the ethnic subgroups, those who were included in the “other Asian” subgroup were mostly recruited outside Asia (45.7%).

### Patterns of OAC prescription

**Fig. 1:**
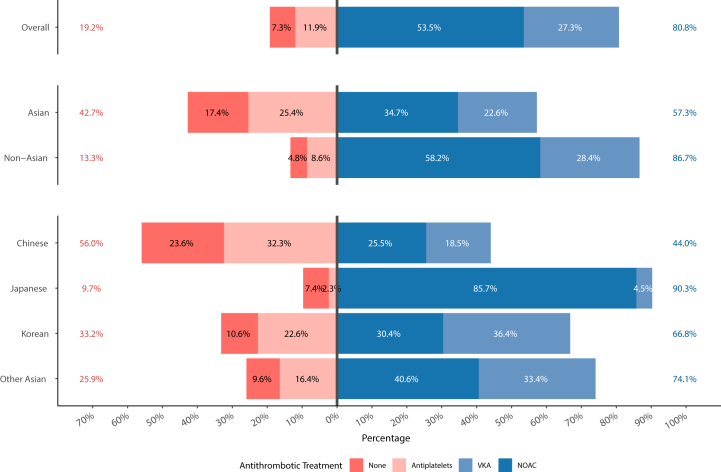
**Rates of antithrombotics prescribed according to Asian ethnicity.** Legend: NOAC = Non vitamin-K antagonist oral anticoagulant; VKA = vitamin K antagonist.

Rates of antithrombotic prescription by ethnicity groups are reported in Fig. 1. Asian patients were less likely prescribed an OAC (57.3% vs. 86.7% in non-Asian), including NOACs (34.7% vs. 58.2%). Consistently, they were more likely not treated with antithrombotic therapy, or treated with antiplatelet drugs. A significant heterogeneity in the pattern of OAC use was observed among Asian ethnicity subgroups, with Japanese patients more frequently treated with OACs (90.3%), specifically NOACs (85.7%), and less likely to receive VKAs, antiplatelet drugs or no antithrombotic treatment; Overall OAC prescription rate was lower in the other ethnic subgroups (74.1% in other Asians, 66.8% in Korean, and 44.0% in Chinese patients). Similar findings were observed for NOAC prescription.

Results of multivariable logistic regression regarding OAC prescription are reported in Table 1. Compared to non-Asian patients, Asian individuals were less likely treated with OAC (OR: 0.23, 95% CI: 0.22–0.25). Similar results were observed (although with different magnitude) for Chinese, Korean, and other Asian patients. Conversely, Japanese patients had higher odds of receiving an OAC (OR: 1.83, 95% CI: 1.41–2.42). Model 2 gave broadly consistent results.

**Table 1:** Multivariable logistic regression on OAC prescription according to Asian ethnicity group and subgroups.

	Asian ethnicity				
	Asian (vs. non-Asian) OR [95% CI]	Chinese (vs. non-Asian) OR [95% CI]	Japanese (vs. non-Asian) OR [95% CI]	Korean (vs. non-Asian) OR [95% CI]	Other Asian (vs. non-Asian) OR [95% CI]
**OAC prescription**					
*Model 1*	**0.23** [**0.22–0.25]**	**0.14** [**0.12–0.15]**	**1.83** [**1.41–2.42]**	**0.37** [**0.33–0.42]**	**0.49** [**0.37–0.65]**
*Model 2*	**0.22** [**0.21–0.24]**	**0.14** [**0.13–0.15]**	**1.66** [**1.27–2.20]**	**0.35** [**0.31–0.39]**	**0.48** [**0.36–0.64]**
**NOAC vs. VKA prescription**					
*Model 1*	**0.66** [**0.61–0.71]**	**0.55** [**0.49–0.61]**	**10.01** [**7.04–14.81]**	**0.39** [**0.34–0.44]**	**0.58** [**0.44–0.77]**
*Model 2*	**0.64** [**0.59–0.69]**	**0.53** [**0.48–0.59]**	**9.51** [**6.68–14.08]**	**0.38** [**0.34–0.43]**	**0.55** [**0.41–0.73]**

Bold text depicts statistically significant results at p < 0.05 level.

Model 1 adjusted for age, sex, type of AF, history of bleeding, BMI, CHA2DS2-VASc score and Phase of Recruitment (Phase III vs. Phase II).

Model 2 adjusted for age, sex, type of AF, history of bleeding, BMI, arterial hypertension, diabetes, history of stroke/TIA, heart failure, PAD, CAD and Phase of Recruitment (Phase III vs. Phase II).

CI = confidence intervals; OAC = oral anticoagulant; OR = odds ratio; NOAC = non-vitamin K antagonist oral anticoagulant; VKA = vitamin K antagonist.

Among those prescribed an OAC, Asian patients were less likely treated with NOAC (OR: 0.66, 95% CI: 0.61–0.71) compared to non-Asians; similar results were observed for Chinese (OR: 0.55, 95% CI: 0.49–0.61), Korean (OR: 0.39, 95% CI: 0.34–0.44) and other Asian (OR: 0.58, 95% CI: 0.44–0.77) subgroups, while Japanese patients showed a higher likelihood of receiving NOAC compared to VKA (OR: 10.01, 95% CI: 7.04–14.81). Model 2 gave similar estimates (Table 1).

Analysing the results according to the enrolment phase (Phase II vs. Phase III), we found lower odds of receiving OAC and NOAC in Asian patients enrolled in Phase II (p for interaction < 0.001 for both). Similar results were observed for OAC prescription in Chinese and Korean patients, with Chinese patients recruited in Phase III, and Korean patients recruited in Phase II less likely to receive NOAC (p < 0.001 for both, Supplementary Fig. S2).

### OAC persistence and discontinuation

Among 27,793 patients who received OAC at baseline, 20,768 (74.7%) had complete follow-up data on OAC discontinuation. Rates of OAC persistence and discontinuation at 6, 12 and 24 months are reported in Supplementary Fig. S3 for Asians vs. non-Asians and Supplementary Fig. S4 for Asian subgroups, respectively.

Cox-regression models showed a higher risk of OAC discontinuation in Asian patients (HR: 1.79, 95% CI: 1.67–1.92), specifically in Chinese (HR: 2.18, 95% CI: 2.00–2.37), Japanese (HR: 1.30, 95% CI: 1.10–1.53) and Korean (HR: 1.58, 95% CI: 1.39–1.80) patients (Table 2). Regression model 2 provided similar estimates. No statistically significant interaction was observed between phase of recruitment and risk of OAC discontinuation in Asian patients (Supplementary Fig. S5). Among Asian ethnicity subgroups, Chinese patients recruited during Phase III, and Korean patients recruited during Phase II were more likely to discontinue OAC (p for interaction = 0.026 and 0.002, respectively) (Supplementary Fig. S6).

**Table 2:** Multivariable Cox regression on OAC discontinuation according to Asian ethnicity.

	Asian ethnicity				
	Asian (vs. Non-Asian) HR [95% CI]	Chinese (vs. non-Asian) HR [95% CI]	Japanese (vs. non-Asian) HR [95% CI]	Korean (vs. non-Asian) HR [95% CI]	Other Asian (vs. non-Asian) HR [95% CI]
**OAC discontinuation**					
*Model 1*	**1.79** [**1.67–1.92]**	**2.18** [**2.00–2.37]**	**1.30** [**1.10–1.53]**	**1.58** [**1.39–1.80]**	1.04 [0.75–1.44]
*Model 2*	**1.81** [**1.68–1.94]**	**2.17** [**1.99–2.37]**	**1.23** [**1.12–1.57]**	**1.62** [**1.42–1.84]**	1.04 [0.74–1.45]
**NOAC discontinuation**					
*Model 1*	**1.89** [**1.73–2.05]**	**2.61** [**2.36–2.90]**	**1.39** [**1.17–1.65]**	**1.48** [**1.27–1.73]**	0.84 [0.53–1.33]
*Model 2*	**1.93** [**1.77–2.10]**	**2.63** [**2.37–2.92]**	**1.42** [**1.19–1.69]**	**1.53** [**1.31–1.79]**	0.83 [0.51–1.34]
**VKA discontinuation**					
*Model 1*	**1.47** [**1.28–1.69]**	**1.41** [**1.21–1.65]**	1.27 [0.48–3.40]	**1.79** [**1.42–2.27]**	1.22 [0.76–1.94]
*Model 2*	**1.44** [**1.25–1.66]**	**1.37** [**1.17–1.60]**	1.22 [0.45–3.26]	**1.79** [**1.42–2.27]**	1.21 [0.76–1.94]

Bold text depicts statistically significant results at p < 0.05 level.

Model 1 adjusted for age, sex, type of AF, history of bleeding, BMI, CHA2DS2-VASc score and Phase of Recruitment (Phase III vs. Phase II).

Model 2 adjusted for age, sex, type of AF, history of bleeding, BMI, arterial hypertension, diabetes, history of stroke/TIA, heart failure, PAD, CAD and Phase of Recruitment (Phase III vs. Phase II).

CI = confidence intervals; HR = hazard ratio; OAC = oral anticoagulant; NOAC = non-vitamin K antagonist oral anticoagulant; VKA = vitamin K antagonist.

The analysis on the risk of OAC discontinuation according to OAC type received at baseline provided broadly similar results (Table 2), although with differences in magnitude of association; no statistically significant differences were observed for Japanese and other Asians patients prescribed with VKA.

### Risk of adverse outcomes

Overall, 24,383 patients (70.8%) had complete follow-up data on the primary outcome and were included in the survival analysis; 4526 (18.6%) were Asian. Compared to patients included, those excluded from this analysis were more likely Asian (p < 0.001) and of female sex (p = 0.017). There were no statistically significant differences in terms of age and proportion of those with high thromboembolic risk (i.e., CHA_2_DS_2_-VASc Score ≥ 2).

**Fig. 2:**
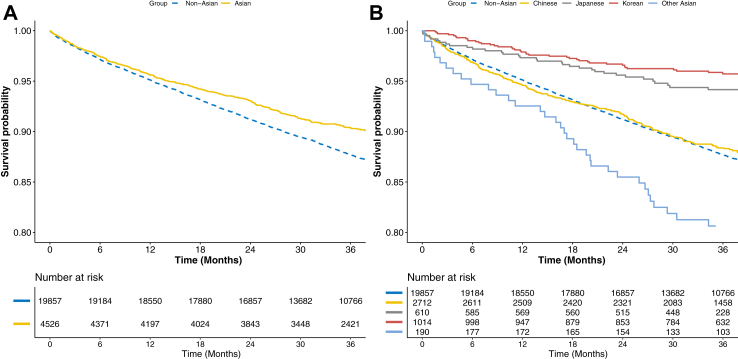
**Survival curves for the primary composite outcome of all-cause death and MACE according to Asian ethnicity.** Legend: Panel A: Asian vs. non-Asian; Panel B: Asian Ethnicity subgroups vs. non-Asian. Log-Rank p < 0.001 for both.

During a median follow-up of 36.2 [IQR: 26.3–37.5] months, a total of 2733 (11.2%) primary composite outcome events were observed. Kaplan–Meier curves for the primary composite outcome according to Asian ethnicity and subgroups are reported in Fig. 2, panels A and B, respectively. Non-Asian patients had a higher incidence of the primary composite outcome compared to Asian individuals (p < 0.001); among the ethnicity subgroups, Japanese and Korean patients showed lower incidence of the primary outcome, while patients in the other Asian subgroups had the highest incidence (p < 0.001).

Multivariable Cox regression analysis showed that Asian ethnicity was associated with a lower risk of the primary composite outcome (HR: 0.86, 95% CI: 0.76–0.96; Table 3). Similar results were observed when comparing Japanese and Korean subgroups with non-Asian patients (HR: 0.53, 95% CI: 0.36–0.78 and HR: 0.42, 95% CI: 0.30–0.59), while no statistically significant differences were observed between Chinese and non-Asian patients (Table 3). Finally, patients in the other Asian subgroup showed a higher hazard of the primary composite outcome (HR: 1.47, 95% CI: 1.06–2.06). Model 2 provided similar estimates.

**Table 3:** Multivariable Cox regressions on the risk of major outcomes according to Asian ethnicity.

	Asian ethnicity					
	non-Asian (n = 19,857)	Asian (n = 4526)	Chinese (n = 2712)	Japanese (n = 610)	Korean (n = 1014)	Other Asian (n = 190)
**Primary outcome**						
Composite of all cause death and MACE						
IR [95% CI]	4.4 [4.3–4.6]	3.5 [3.1–3.8]	4.2 [3.8–4.7]	2.1 [1.4–2.9]	1.5 [1.0–2.0]	7.5 [5.3–10.3]
Model 1, HR [95% CI]	Ref.	**0.86** [**0.76–0.96]**	1.00 [0.87–1.14]	**0.53** [**0.36–0.78]**	**0.42** [**0.30–0.59]**	**1.47** [**1.06–2.06]**
Model 2, HR [95% CI]	Ref.	**0.84** [**0.74–0.94]**	0.93 [0.81–1.07]	**0.56** [**0.38–0.82]**	**0.45** [**0.32–0.63]**	**1.65** [**1.18–2.32]**
**Secondary outcomes**						
All cause death						
IR [95% CI]	3.5 [3.3–3.6]	2.1 [1.8–2.3]	2.7 [2.3–3.0]	1.1 [0.6–1.7]	0.7 [0.4–1.1]	4.3 [2.7–6.5]
Model 1, HR [95% CI]	Ref.	**0.67** [**0.58–0.78]**	**0.83** [**0.70–0.98]**	**0.33** [**0.19–0.56]**	**0.26** [**0.16–0.42]**	1.04 [0.68–1.61]
Model 2, HR [95% CI]	Ref.	**0.66** [**0.56–0.76]**	**0.77** [**0.65–0.91]**	**0.34** [**0.20–0.59]**	**0.28** [**0.17–0.45]**	1.25 [0.81–1.93]
CV death						
IR [95% CI]	1.2 [1.1–1.3]	0.9 [0.8–1.1]	1.2 [1.0–1.5]	0.3 [0.1–0.7]	0.3 [0.1–0.6]	2.0 [0.9–3.6]
Model 1, HR [95% CI]	Ref.	0.91 [0.72–1.14]	1.13 [0.88–1.46]	**0.38** [**0.16–0.92]**	**0.34** [**0.16–0.73]**	1.36 [0.72–2.56]
Model 2, HR [95% CI]	Ref.	0.88 [0.70–1.11]	1.02 [0.78–1.32]	**0.40** [**0.17–0.98]**	**0.39** [**0.18–0.83]**	1.81 [0.96–3.42]
MACE						
IR [95% CI]	2.2 [2.1–2.4]	2.3 [2.0–2.6]	2.7 [2.4–3.1]	1.4 [0.9–2.2]	1.1 [0.7–1.5]	5.3 [3.5–7.8]
Model 1, HR [95% CI]	Ref.	1.13 [0.97–1.32]	**1.27** [**1.06–1.51]**	0.82 [0.53–1.27]	**0.63** [**0.43–0.93]**	**2.06** [**1.39–3.04]**
Model 2, HR [95% CI]	Ref.	1.11 [0.95–1.29]	1.18 [0.98–1.41]	0.87 [0.56–1.35]	0.68 [0.46–1.00]	**2.35** [**1.59–3.50]**
Thromboembolism						
IR [95% CI]	1.2 [1.1–1.3]	1.6 [1.3–1.8]	1.8 [1.5–2.1]	1.2 [0.7–1.9]	0.8 [0.5–1.2]	3.9 [2.3–6]
Model 1, HR [95% CI]	Ref.	**1.33** [**1.10–1.61]**	**1.42** [**1.14–1.77]**	1.16 [0.72–1.90]	0.76 [0.48–1.21]	**2.79** [**1.75–4.44]**
Model 2, HR [95% CI]	Ref.	**1.32** [**1.09–1.60]**	**1.42** [**1.14–1.78]**	1.18 [0.73–1.93]	0.76 [0.48–1.21]	**2.45** [**1.53–3.92]**
Major bleeding						
IR [95% CI]	1.3 [1.2–1.4]	0.8 [0.6–1.0]	0.7 [0.5–0.9]	1.3 [0.8–2]	0.5 [0.3–0.8]	2.0 [0.9–3.6]
Model 1, HR [95% CI]	Ref.	**0.77** [**0.61–0.98]**	**0.68** [**0.50–0.93]**	1.24 [0.77–1.99]	**0.54** [**0.31–0.94]**	1.39 [0.72–2.70]
Model 2, HR [95% CI]	Ref.	**0.77** [**0.60–0.98]**	**0.65** [**0.48–0.90]**	1.31 [0.81–2.10]	**0.56** [**0.32–0.98]**	1.53 [0.78–2.97]
Intracranial haemorrhage						
IR [95% CI]	0.3 [0.2–0.3]	0.4 [0.3–0.5]	0.4 [0.2–0.5]	0.3 [0.1–0.7]	0.3 [0.1–0.6]	1.0 [0.3–2.3]
Model 1, HR [95% CI]	Ref.	**1.54** [**1.04–2.26]**	1.45 [0.90–2.33]	1.42 [0.57–3.52]	1.36 [0.65–2.84]	**3.39** [**1.37–8.41]**
Model 2, HR [95% CI]	Ref.	**1.42** [**0.96–2.10]**	1.32 [0.81–2.15]	1.34 [0.54–3.32]	1.27 [0.61–2.67]	**3.13** [**1.25–7.84]**
Non ICH major bleeding						
IR [95% CI]	1.0 [1.0–1.1]	0.4 [0.3–0.6]	0.4 [0.2–0.5]	1.0 [0.6–1.6]	0.2 [0.1–0.4]	1.0 [0.3–2.3]
Model 1, HR [95% CI]	Ref.	**0.51** [**0.37–0.71]**	**0.43** [**0.28–0.66]**	1.14 [0.66–1.99]	**0.27** [**0.11–0.66]**	0.79 [0.30–2.13]
Model 2, HR [95% CI]	Ref.	**0.53** [**0.38–0.73]**	**0.42** [**0.27–0.65]**	1.24 [0.71–2.17]	**0.29** [**0.12–0.71]**	0.92 [0.34–2.47]

Bold text depicts statistically significant results at p < 0.05 level.

Model 1 adjusted for age, sex, type of AF, history of bleeding, BMI, use of OAC, CHA2DS2-VASc score and Phase of Recruitment (Phase III vs. Phase II).

Model 2 adjusted for age, sex, type of AF, history of bleeding, BMI, use of OAC, arterial hypertension, diabetes, history of stroke/TIA, heart failure, PAD, CAD and Phase of Recruitment (Phase III vs. Phase II).

CI = confidence intervals; HR = hazard ratio; IR = incidence rate; Ref = reference.

### Exploratory secondary outcomes

Asian ethnicity group was associated with lower risk of all-cause death (HR: 0.67, 95% CI: 0.58–0.78), major bleeding (HR: 0.77, 95% CI: 0.61–0.98) and non-ICH major bleeding (HR: 0.51, 95% CI: 0.37–0.71), and with higher risk of thromboembolism (HR: 1.33, 95% CI: 1.10–1.61), and ICH (HR: 1.54, 95% CI: 1.04–2.26) (Table 3). Among the Asian ethnicity subgroup, Korean and Japanese patients had the lowest risk of all-cause and cardiovascular death, with Korean patients also showing a lower risk of major bleeding and non-ICH major bleeding. Patients in the other Asian subgroup had a higher risk of MACE, thromboembolism and major bleeding.

The results on the interaction between phase of recruitment, Asian ethnicity and risk of adverse outcomes are reported in Supplementary Fig. S7. No statistically significant interactions were observed for the outcomes investigated, for both regression models; however, Asian patients recruited in Phase III showed lower risk of both major bleeding (p for interaction = 0.091) and non-ICH major bleeding (p for interaction = 0.070).

## Discussion

In this analysis, based on a global contemporary, prospective cohort of patients with AF, we showed: 1) patterns of OAC prescription were different between non-Asian and Asian patients, with the latter more frequently undertreated with OAC and NOAC; 2) significant differences in OAC prescription between subgroups of Asian patients, with Japanese more likely to receive OAC and NOAC, and the other subgroups more often undertreated; 3) the risk of OAC discontinuation during follow-up was higher among Asian patients with AF, with some differences observed among Asian ethnicity subgroups; 4) Asian patients had an heterogeneous risk of adverse outcomes, with our results showing associations with lower risks of the primary composite outcome, death and major bleeding, but with a higher risk of thromboembolic events and ICH; and 5) risks were heterogeneously distributed among ethnically Asian subgroups, suggesting that further differences may be present.

The increasing uptake of NOAC has changed the landscape of antithrombotic risk prevention worldwide. However, geographical differences in pattern of OAC use have already been described, in GLORIA-AF Registry and other studies, and undertreatment of Asian patients has been reported.^13^^,^^14^^,^^20^ Also, Asian patients have been described at higher risk of both thromboembolism and ICH, thus underlining the high risk of adverse outcomes, and the unmet need for better management of these patients.^11^^,^^21^

In this analysis, we found that Asian patients were overall less prescribed an OAC compared to non-Asian individuals (which were mainly recruited in Europe and North-America). Moreover, we found significant heterogeneity among Asian subgroups, with Japanese patients receiving an OAC more frequently, and with a 10-fold higher odds of receiving a NOAC over a VKA. We found that Asian patients (including Japanese) were more likely to discontinue OAC during follow-up, suggesting that determinants of OAC underprescription may also influence the risk of discontinuation in Asian patients.

Several reasons can explain these findings, and disentangling the effect of ethnicity to those of the region or country of recruitment is challenging. Different timings in market authorisation of NOACs between countries, and different healthcare policies regarding reimbursement and costs can explain, at least partly, differences observed between Asian and non-Asian patients, and in Asian subgroups.^22^ For example, NOACs are self-paid in China,^23^ and willingness to pay has been shown to be heterogeneous according to educational level and income of patients, thus potentially influencing both OAC prescription and persistence.^24^ Nonetheless, previous evidence has shown that Asian (as well as Black) patients with AF were less likely than White patients to initiate OAC, even in the same geographical setting.^25^^,^^26^ Also, prescription of lower-dose NOAC is common among Asian patients; the latter may expose them to a higher risk of unfavorable prognosis when dose-reduction criteria are not met.^27^^,^^28^ Potential reasons for these differences could reside in different perceptions, among treating physicians, of the baseline thromboembolic and haemorragic risks of different ethnicities, as well as in imbalances in the access to care.^25^ While these findings seem consistent with our results (and with the potential ethnicity-based differences in OAC prescription), whether our findings can be wholey attributed to the influence of ethnic or geographical factors (or to the contribution of both) remains an open question, and will require further investigation.

Indeed, differences between Asian ethnicities (such as Chinese and other Asian) were reported in the GARFIELD-AF registry, showing how Chinese patients were more likely treated with VKA.^29^ Of note, geographical differences in quality of anticoagulation have already been reported, with previous study showing low proportion of VKA-treated Asian patients with good time in therapeutic range.^30^ Finally, we observed some imbalances in thromboembolic risk factors between different Asian ethnicities: while Japanese patients exhibited similar age and proportion of patients with high CHA_2_DS_2_-VASc score compared to non-Asians, other ethnicity subgroups were overall younger, and had lower baseline thromboembolic risks. All these factors could also have contributed to OAC prescription and discontinuation in our study.

Taken together, all these factors can explain the different patterns of OAC prescription and discontinuation observed in Asian patients in our study. Of note, OAC utilization has been increasing over last decade in Asian countries, particularly driven by higher adoption of NOACs.^31^, ^32^, ^33^ This is consistent with our results on the interaction between phase of recruitment and odds of OAC and NOAC prescription, considering that Phase III of the GLORIA-AF registry was conducted after the Phase II.

Our findings suggest that, while unsatisfactory OAC treatment is still a global concern, this may represent a concern that disproportionately affects Asian patients. This is important given that our results also show that Asian patients with AF may present a specific risk profile in terms of major outcomes: we found a potential association between Asian ethnicity and higher risk of thromboembolic events and ICH, with also lower risk of all-cause death and overall major bleeding (mostly driven by non-ICH major bleeding).

While these results are in line with previous evidence, which showed a higher risk of stroke/thromboembolism and ICH in Asian patients,^12^^,^^34^ the association between Asian ethnicity and lower risk of major bleeding, and specifically non-ICH major bleedings raises several hypotheses. First, this may reflect the higher uptake of NOAC in the Asian populations compared to previous studies, as the risk reduction in major bleeding observed with NOACs was higher in Asians compared to non-Asian patients.^1^^,^^12^^,^^35^ Second, the higher risk of thromboembolism, paired with the overall lower risk of major bleeding, may not be due only to ethnic-specific predisposition, but also to the lower OAC use among Asians patients, as in our cohort. Third, despite the overall lower risk of major bleeding, Asians individuals were still found at higher risk of ICH, similar to previous observations.^34^ This is consistent with the hypothesis of specific ethnic-factors which may predispose to ICH, including a potentially higher prevalence of cerebral microbleeds and small cerebral vessel disease, as well as genetic polymorphisms associated to bleeding.^36^ Finally, the lower risk of mortality found in Asian patients may be due to differences in baseline characteristics and risk factors between Asian and non-Asian patients. Overall, also considering the exploratory nature of these associations, these results need confirmation in other cohorts and clinical scenarios, and should therefore interpreted with caution.

Taken together, our results have several clinical implications. Given the unsatisfactory OAC treatment (in terms of prescription and persistence), further efforts should be dedicated to ensure that Asian patients with AF receive adequate thromboembolic risk prevention. This appears particularly important when considering their higher risk of both thromboembolism and ICH. Indeed, patients with AF require careful management to improve their long-term prognosis and the ‘Atrial fibrillation Better Care’ (ABC) pathway has been proposed to streamline a holistic and integrated care approach to the management of patients with AF, encompassing rational choice regarding OAC, patient-centred strategies to achieve better symptoms control, and optimal management of comorbidities and risk factors.^37^ This approach has been already proved effective in reducing the risk of adverse outcomes,^38^^,^^39^ including thromboembolism and major bleedings.^40^ Moreover, its efficacy has been specifically tested and proven in Asian settings,^41^ including a cluster randomised trial performed in China.^42^ Nonetheless, adherence to the ABC pathway has been found to be unsatisfactory,^40^ and given the findings of our study, its large-scale application should be encouraged. Indeed, the latest Asia Pacific Heart Rhythm Society Guidelines for the management of patients with AF recommended the implementation of the ABC pathway in clinical practice, thus reinforcing how these patients require an holistic and integrated approach to improve their outcomes.^43^

Our study also leaves several open questions. Among the others, whether the results observed can be attributed clearly to ethnicity rather than other factors (geographical or country-specific characteristics, socioeconomic status, differences in local practice, healthcare systems, and reimbursement policies). Interestingly, recent US-based analyses have suggested that differences between Asian and non-Asian patients may be mitigated when they are treated in the same geographical setting.^44^^,^^45^ Further studies are therefore needed to disentangle the effect of ethnicity (e.g., the perceived higher risk of ICH in Asians which may influence OAC prescription) to that of the country and geographical region.^46^

Our study represents a large comparison of Asian and non-Asian individuals, based on a contemporary, global cohort of newly-diagnosed patients with AF and using self-reported ethnicity. The findings are largely consistent with previous, preliminary observations on the topic, and contribute to expand our knowledge on the potential ethnic differences in AF. Furthermore, we reported on OAC prescription and discontinuation, as well as major outcomes, thus offering us a comprehensive outlook on the management and natural history of patients with AF.

Nonetheless, our study has some limitations. First, this is a post-hoc analysis of an observational prospective study, and therefore we may have limited power to detect differences, especially in some Asian ethnic subgroups (for which our results should be regarded as hypothesis-generating). The analysis is based on self-reported ethnicity, and a small proportion of patients were excluded due to lack of data. Other factors may have influenced patients’ choice regarding their self-reported ethnicity, which we were unable to explore. Specifically, we were only able to analyse data according to the region of recruitment and not country, and therefore we could not analyse country-specific differences in terms of healthcare systems and reimbursement policies, and these factors may exert an important role in determining the differences observed, especially between Asian subethnicities. Moreover, we cannot fully exclude the effect of unaccounted confounders in the relationship between Asian ethnicity and outcomes (including OAC prescription and discontinuation); specifically, it is possible that imbalance in clinical characteristics, country-specific factors (most Asian patients were recruited in Asian countries), socio-economical factors, and timing of recruitment may have influenced the results observed, which should therefore interpreted with caution. However, we provided both interaction analysis according to recruitment phase, and two different covariate-adjusted models, which gave broadly similar results. We considered switching to another antithrombotic regimen as an OAC discontinuation in this analysis, consistently with previous analysis on the same registry,^47^ and this may have influenced the associations observed between ethnicity and OAC discontinuation; moreover, the imbalance in OAC discontinuation during follow-up could have influenced in turn the association between ethnicity and outcomes. Further studies are clearly required to address this issue. Finally, our results on secondary outcomes (including thromboembolism and major bleeding events) were not adjusted for multiple comparisons, and therefore should be interpreted with caution.

In this large prospective global registry of patients with AF, Asian patients were less likely prescribed with OAC and NOAC, with some heterogeneity between different ethnic subgroups. Moreover, Asian ethnicity was associated with OAC discontinuation, and lower risk of the composite outcome of all-cause death and MACE. Among exploratory secondary outcomes, Asian ethnicity was associated with lower risk of major bleeding, but higher risks of thromboembolic events and ICH. Improvements in the management of AF are needed to improve outcomes in Asian patients with AF.

## Contributors

GFR, BC, and GYHL conceived and designed the analysis, and verified the underlying data; GFR analysed data; GFR and BC interpreted data and drafted the manuscript; MP, DAM, JF, AB, GB, BO, YHC, MVH, TFC, and GYHL revised the manuscript and gave relevant intellectual contribution. GFR, BC, and GYHL had access to the dataset, all authors read and approved the final manuscript, and accept responsibility to submit for publication.

## Data sharing statement

Data supporting this study by the data contributors Boehringer Ingelheim, and were made and are available through Vivli, Inc. Access was provided after a proposal was approved by an independent review committee identified for this purpose and after receipt of a signed data sharing agreement.

## Declaration of interests

**GFR** reports consultancy for Boehringer Ingelheim and an educational grant from Anthos, outside the submitted work. No fees are directly received personally. **AB** has been a consultant or speaker for Astra-Zeneca, Bayer, BMS/Pfizer, Medtronic, Vitorpharma and Alnylam. **TFC** reported honoraria for lectures from Boehringer Ingelheim, Bayer, Pfizer, and Daiichi Sankyo, outside the submitted work. **GB** reports small speaker fees from Bayer, Boehringer Ingelheim, Boston, BMS, Daiichi, Sanofi, Janssen outside the submitted work. **MVH** has been receiving research grants from the Dutch Healthcare Fund, Dutch Heart Foundation, BMS-Pfizer, Bayer Healthcare and Boehringer Ingelheim and consulting fees from BMS-Pfizer, Bayer Healthcare and Boehringer Ingelheim to the institution. All other authors have nothing to declare. **GYHL** has been consultant and speaker for BMS/Pfizer, Boehringer Ingelheim, Anthos and Daiichi-Sankyo. No fees are directly received personally. All the disclosures happened outside the submitted work. **GYHL** is co-principal investigator of the AFFIRMO project on multimorbidity in AF, which has received funding from the European Union’s Horizon 2020 research and innovation programme under grant agreement No 899871.
